# Microstructures and Macrosegregation of Al–Zn–Mg–Cu Alloy Billet Prepared by Uniform Direct Chill Casting

**DOI:** 10.3390/ma14040708

**Published:** 2021-02-03

**Authors:** Li Zhou, Yajun Luo, Zhenlin Zhang, Min He, Yinao Xu, Yulei Zhao, Sheng Liu, Lijun Dong, Zhifeng Zhang

**Affiliations:** 1 Hunan Engineering Research Center of New Energy Vehicle Lightweight, Hunan Institute of Engineering, Xiangtan 411104, China; zhouli@hnie.edu.cn (L.Z.); xya@stu.hnie.edu.cn (Y.X.); z0222ddda@stu.hnie.edu.cn (Y.Z.); 18028@hnie.edu.cn (S.L.); dlj@hnie.edu.cn (L.D.); 2 Wenchang New Material Technology Co. Ltd., Loudi 417000, China; marketing@hwtc.cc (Z.Z.); hemin@hwtc.cc (M.H.); 3 General Research Institute for Non-Ferrous Metals, Beijing 100088, China; zhangzf@grinm.com

**Keywords:** Al–Zn–Mg–Cu alloy, direct chill casting, electromagnetic stirring, intercooling, grain refinement, macrosegregation

## Abstract

In this study, large-sized Al–Zn–Mg–Cu alloy billets were prepared by direct chill casting imposed with annular electromagnetic stirring and intercooling; a process named uniform direct chill casting. The effects of uniform direct chill casting on grain size and the alloying element distribution of the billets were investigated and compared with those of the normal direct chill casting method. The results show that the microstructures were refined and the homogeneity of the alloying elements distribution was greatly improved by imposing the annular electromagnetic stirring and intercooling. In uniform direct chill casting, explosive nucleation can be triggered, originating from the mold wall and dendrite fragments for grain refinement. The effects of electromagnetic stirring on macrosegregation are discussed with consideration of the centrifugal force that drives the movement of melt from the central part towards the upper-periphery part, which could suppress the macrosegregation of alloying elements. The refined grain can reduce the permeability of the melt in the mushy zone that can restrain macrosegregation.

## 1. Introduction

The Al–Zn–Mg–Cu alloy with a high strength-to-density ratio, considerable fracture toughness and good stress corrosion cracking resistance has been widely used for military and commercial applications [1,2,3,4,5,6]. Despite its positive characteristics, the main bottleneck for this alloy is how to prepare large-sized high quality billets or ingots for final applications of forging or extrusion. With the increase in billet size, permanent-mold casting and direct chill (DC) casting methods have been developed successively to increase the cooling rate. At present, DC casting is the main way to obtain billets [7,8]. However, during the process of DC casting, the melt can only be cooled and solidified from outside to inside by the mold cooling (primary cooling) and by spraying water cooling (secondary cooling). Due to the long distance from the edge to the center of the billet for heat conduction, the effect of secondary cooling cannot be fully brought into play [9]. Therefore, as the billet size increases, it is more and more difficult to obtain the uniformity of the melt temperature field. Especially in the primary cooling area that is in contact with the graphite ring, the non-uniform melt temperature leads to a non-uniform solidification shell which exhibits remarkable thermal stresses and strains, and is prone to wrinkles, segregation tumors and even leaks of billets [10,11]. To reduce the heat extraction rate and the thermal strain during the DC casting process, the wiper is implemented to divert the free falling water from the billet surface, thus, drastically reducing the heat extraction rate [12]. Due to the application of the wiper, the billet pulling speed should be reduced to ensure that billets have enough time to solidify. In addition, the large volume melt solidification will release a lot of latent heat that takes a considerable amount of time for the melt center to cool. Therefore, in order to ensure the smooth progress of the DC casting process, the pulling speed should be reduced to ensure that the heat of the melt itself and the latent heat of solidification are fully released. However, if the casting speed is too slow, there is enough time for the nuclei to grow up, meanwhile, the formed nuclei would be dissolved again, resulting in the reduction in nucleation sites. As a result, the microstructure of the billet is non-uniform and coarse.

Recently, many researchers have studied the application of external force including mechanical stirring, ultrasonic vibrating and electromagnetic stirring on DC casting to improve the uniformity of the temperature field of billets and to obtain high quality billets with fine and uniform structures [13,14,15,16]. Electromagnetic stirring (EMS) is the most widely used melt treatment technology in industry and is widely used in the field of steel and non-ferrous metal continuous casting [17,18]. However, due to the skin effect, electromagnetic stirring technology is limited in the preparation of large-sized aluminum alloy billets. For this reason, the previous research group put forward Annular Electromagnetic Stirring (A-EMS), which provided a new idea to solve the skin effect [19]. The mechanism of these methods is to enhance the thermal convection of the melt to improve the uniformity of the temperature field, but the single cooling mode that the melt was only cooled by the mold and spraying water is not changed, the temperature difference between the edge part and the center part of the billets is still remarkable [20]. From the point of view of the development of large size billet preparation technology, this is mainly achieved through improving the cooling rate of the melt from mold cooling alone to mold cooling and spray water cooling. Therefore, based on A-EMS technology, a uniform direct chill (UDC) casting method was designed for extracting heat from the melt center and preparing large-sized billets by coupling A-EMS with intercooling in the sump during the DC casting process [21]. In previous studies, the effects and mechanism of UDC on microstructures, macrosegregation and the mechanical properties of the 7005 aluminum alloy were investigated and discussed [20,22]. However, the content of the alloy elements of the 7005 aluminum alloy is relatively low, which is not sufficient to illustrate that UDC casting can be applied to prepare other large-sized 7xxx series high strength aluminum alloy billets containing high alloying elements.

In this paper, the recently developed UDC casting was employed for the production of large-sized Al–Zn–Mg–Cu alloy billets with improved quality, and the operating mechanisms responsible for the microstructure refinement and composition homogenization were analyzed in detail.

## 2. Experimental

**Figure 1:**
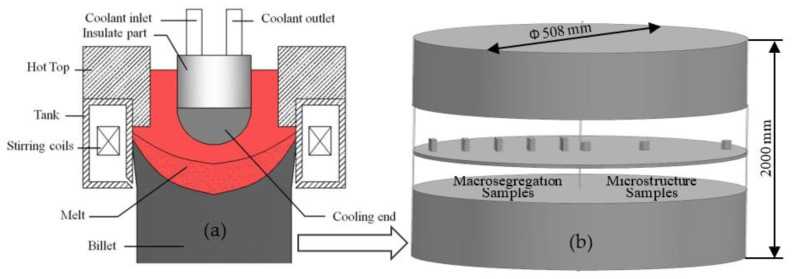
Schematic illustration of the uniform direct chill (UDC) casting process (**a**) [21] and sampling location of billet for structural inspection and chemical composition measurement (**b**).

The schematic illustration of the UDC casting is shown in Figure 1 [21]. Based on the normal direct chill (NDC) casting equipment, the intercooling is realized by an in-mold cooler with a cooling end made of highly pure graphite placed in the central axis of the mold. Thus, there was annular gap between the in-mold cooler and the mold, electromagnetic stirrer arranged with six water-cooled copper coils fed with a three-phase electric current was externally installed around the mold to achieve A-EMS.

Commercial pure aluminum (99.85 wt.%), commercial pure zinc (99.995 wt.%), commercial pure magnesium (99.90 wt.%), commercial pure copper (99.97 wt.%), Al-4Zr master alloys master alloy were melted in an industrial induction furnace (Idea electric co., LTD., Shijiazhuang, China) to obtain the experimental Al–Zn–Mg–Cu alloy without grain refiner. It should be indicated that the composition of the alloy is Al–5.91 Zn–2.32 Mg–2.26 Cu–0.13 Zr–0.072 Fe–0.031 Si (wt.%). After being degassed and filtered, the molten alloy whose temperature reached 1023 K was poured into the preheated hot top of a DC caster with a billet diameter of 508 mm. The intercooling and A-EMS were started at the same time when the casting process proceeded at a stable stage. The NDC and UDC casting process parameters are shown in Table 1.

To avoid cracking, the billets were homogenized at 743 K for 24 h immediately after being produced by the NDC casting and UDC casting, and then air-cooled to room temperature. For structural inspection and chemical composition measurements, the samples were sliced along its half-length, as shown in Figure 1b, where the casting process is steady. The samples were deposited with anodic coatings at 30 V direct current in 2.5 vol.% fluoroboric acid solution. A polarized light optical microscope equipped with an image analysis system (Carl Zeiss Axiovert 200 MAT, Carl Zeiss AG, Heidenheim and der Brenz, Germanywas used for metallography observations. The chemical composition was determined by a Foundry-Master Pro direct reading spectrometer (Oxford Instruments, Taunusstein, Germany), five chemical composition measurements were made to obtain the average of each condition.

## 3. Results

**Figure 2:**
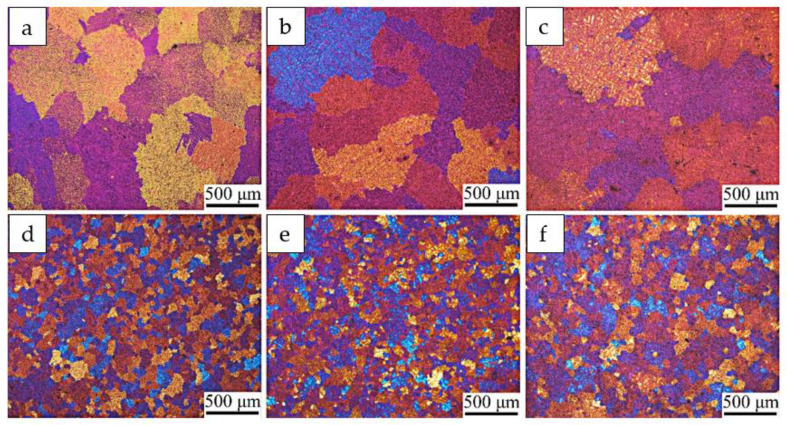
Micrographs of radial cross sections of the edge (**a**,**d**), the 1/2 radius (**b**,**e**) and the center (**c**,**f**) of the Φ508 mm Al–Zn–Mg–Cu alloy billets prepared by NDC casting (**a**–**c**) and UDC casing (**d**–**f**).

**Figure 3:**
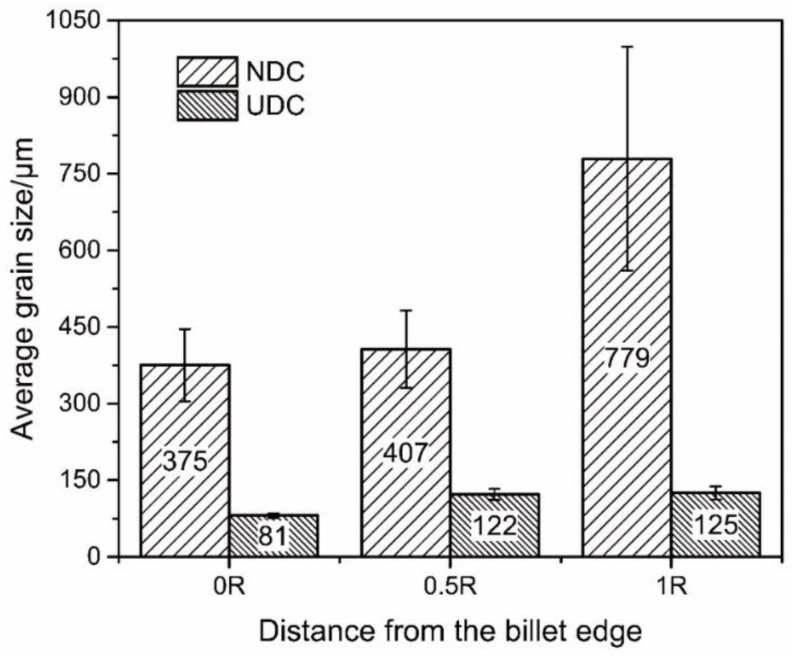
Data indicating the average grain size along the radius direction of the billets formed using NDC casting.

Figure 2 presents the micrographs of the Al–Zn–Mg–Cu alloy billets prepared by NDC casting and UDC casting, and Figure 3 shows the data for the average billet grain size along the radius direction of the billets formed using NDC casting and UDC casting. Dramatic discrepancies in the microstructure between the two different casting billets were observed. The microstructure throughout the Al–Zn–Mg–Cu alloy billet, prepared by NDC casting, is typically made of coarse grains. These coarse grains are developed dendrites that are distributed all over the billets. The average grain size of the billet increases from 375 μm in the periphery section to 779 μm in the central section due to the lower cooling rate in the central section. Therefore, it is difficult to prepare large-sized Al–Zn–Mg–Cu alloy billets with fine and uniform microstructure by NDC casting. It can be noted that the whole microstructure of the billet prepared by UDC casting was fine equiaxed grains. The average grain size in the periphery part of the billet was only 81 μm. Owing to intercooling, the grain size in the central part of the billet only increased a little to 125 μm. It was calculated that the average grain size decreased from 520 ± 110 μm to 109 ± 10 μm by using UDC.

To evaluate the macrosegregation in the radius direction of billet, it is necessary to measure the main alloying elements along the radius direction of the billets. Relative segregation, which is defined by the following equation, is used to describe the degree of macrosegregation.

(1) $$C=\left(C_{i}-C_{0}\right)/C_{0}$$

where, *C_i_* is the measured content of element *i*, *C*_0_ is the nominal content of the element.

**Figure 4:**
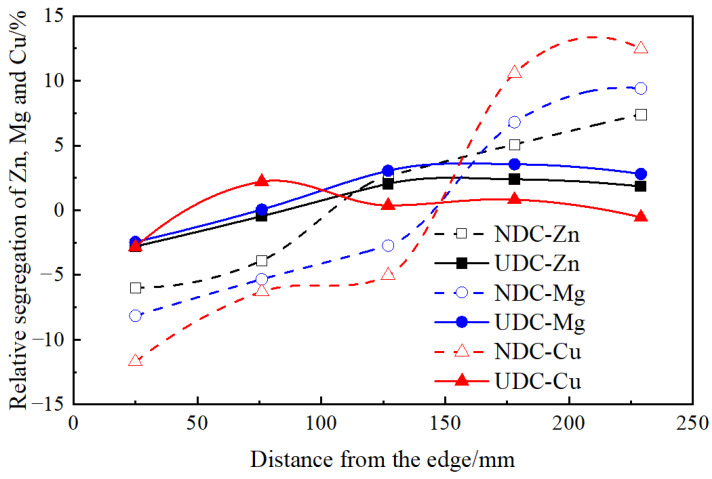
Zn, Mg and Cu elements distribution along radical direction of Φ508 mm Al–Zn–Mg–Cu alloy billets prepared by NDC casing and UDC casting.

Figure 4 shows the relative segregation of the main alloying elements along the radius direction of the Al–Zn–Mg–Cu alloy billets with diameter of 508 mm, prepared by NDC and UDC casting. It is apparent that all the element segregation patterns are the same for the billet prepared by NDC or UDC casting, but the degree of segregation varies greatly between the two different casting billets. This demonstrates the typical negative surface segregation, positive mid-radius segregation and positive centerline segregation for the NDC or UDC casting billets. In the NDC casting process, the natural thermo-solutal convection facilitates negative surface segregation and positive centerline segregation of Zn, Mg and Cu. For example, the negative surface segregation of Zn, Mg and Cu is enhanced to 6, 8 and 12%, respectively. The forced flow in the UDC casting process can effectively eliminate macrosegregation, and the deviation of Zn, Mg and Cu is less than 3%.

## 4. Discussion

In the UDC casting process, the occurrence of explosive nucleation is essential for effective grain refinement. Two different routes that exist in UDC casting are responsible for explosive nucleation. The nuclei could originate from the graphite ring wall and in-mold cooler wall. Meanwhile, the forced flow can break or fuse the dendrites into fragments that can be brought to the remaining liquid and finally form nuclei. These will be discussed in detail as below.

**Figure 5:**
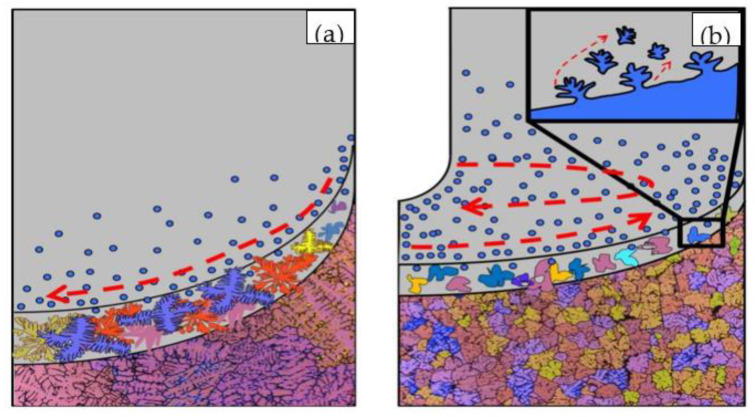
The Schematic diagram showing grain nucleation and growth of NDC (**a**) and UDC (**b**) casting.

Both the intercooling and the A-EMS have a certain effect on grain refinement, and the UDC casting technology makes full use of the advantages of both. The implementation of an in-mold cooler can remove heat from the sump center. Intercooling works with the mold cooling and spraying water cooling to reduce the heat energy of the large volume melt in the sump for preparing large-sized billet. Meanwhile, the A-EMS can contribute to the heat convection of the melt in the sump. Due to the effect of intercooling and A-EMS, the temperature of the melt in the billet center decreases significantly during the UDC casting process compared to the NDC casting [21]. Therefore, the cooling rate of the melt can be significantly improved by the UDC casting. Numerous nuclei were formed alongside the graphite ring in the sump during the NDC and UDC casting processes, owing to the rapid cooling rate of the melt near the mold (as shown in Figure 5). In addition, the melt also can nucleate around the in-mold cooler in the UDC casting process. Therefore, the nucleation rate of the UDC is higher than that of the NDC. In the NDC casting process, these nuclei can only be driven to move down along the sump slope by the weak natural thermo-solutal convection of the melt. This is shown in Figure 5a with just a few nuclei flowing to the sump center and growing up to the coarse dendrite under the conditions of enough space and time. However, under the effect of A-EMS in the UDC casting process, all the nuclei formed around the graphite ring and in-mold cooler can be brought to anywhere in the sump by the forced flow, as shown in Figure 5b. These nuclei are dispersed homogeneously throughout the sump and nuclei growth is blocked by each other in the limited space.

Another mechanism plays the role of grain refinement in the UDC casting process. A-EMS could fuse or break the dendrites into fragments that can be melted and become new nuclei. On the one hand, under the effect of A-EMS on the melt, a Lorentz force is generated, which can break the formed dendrite. Campanella et al. [23] gives the criterion of dendrite fracture in electromagnetic stirring DC casting process:

(2) $$C_{R}\approx \frac{1}{v}\frac{K}{g_{l}\cdot \mu }\frac{B_{0}^{2}}{\mu_{0}d_{ind}}>1$$

where, $g_{l}$, *μ* and $\mu_{0}$ refer to the volume fraction of the liquid, the dynamic viscosity of the melt and the vacuum permeability, respectively. Additionally, *K*, dind and $B_{0}$ refer to the permeability of mushy zone, the distance between the inductor and the liquidus front and the magnetic field, respectively. v is the casting speed. It can be seen from the above formula that the dendrite fracture will occur as long as the relationship between the magnetic induction strength and the melt meets the above requirements. The value of *C_R_* at the radius of 1/2 of the graphite ring in the process of UDC casting is 1.61. Liotti et al. [24] demonstrated that the electromagnetic field can break dendrites into fragments by in situ synchrotron X-ray radiography. On the other hand, the electromagnetic stirring accelerates the melt convection, resulting in the high-temperature melt reaching the dendrite root. The arms of a columnar or dendrite remelt at their necks and form fragments [25]. Therefore, the dendrite in the UDC casting can be broken or fused into fragments, as shown in the enlarged part of Figure 5b. The fragments could be driven to the sump central part as new nucleation sites [26,27]. Under the combined action of electromagnetic stirring and intercooling, explosive nucleation appears throughout the mushy zone. Nuclei compete with each other to grow up, resulting in a fine and uniform microstructure.

In the NDC casting, the melt is mainly driven by the temperature buoyancy force and moves down along the sump slope to the sump center, leading to the solute-rich melt being formed in the upper-periphery part of the mush flows to the lower-central mush, as shown in Figure 5a. Therefore, negative segregation occurs at the surface and positive segregation occurs in the central part of the billet [28]. However, due to the increase in the melt cooling rate and the improvement in the temperature field uniformity in the UDC casting, the sump is obviously shallower than that in the NDC casting [20,29]. The sump slope in the UDC casting is more gradual than that in the NDC casting, the solute-rich melt that flows along the slope from the upper-periphery part towards the central part becomes difficult in the sump during the UDC casting process. Moreover, it is considered that the centrifugal force field is presented in the electromagnetic stirring. Under the action of the centrifugal force, the solute-rich melt tends to do an outwards radial motion, in addition, to a rotary motion. Yao et al. [30] also found that electromagnetic stirring generated a centrifugal movement, which brought high temperature melt from the center of molten pool to its edge. Therefore, the temperature buoyancy force can be offset by the centrifugal force, slowing down the flow velocity of solute-rich melt flow from upper-periphery part towards the central part. The negative surface segregation and positive centerline segregation can be relieved.

Grain size is another factor that has an influence on macrosegregation. During the later stages of the solidification process, the dendrites can bridge each other and form a continuous solid network. Solute-rich melt flows through channels of that solid network. The depth of melt penetration can be described by permeability, calculated as [31]:

(3) $$K=\frac{d^{2}{\left(1-f_{s}\right)}^{3}}{180f_{s}^{2}}$$

where $f_{s}$ is the solid fraction, and d is the grain size. The average grain size of the billet prepared by NDC casting and UDC casting is 520 and 109 μm, respectively. The permeability of UDC casting is lower than that of NDC casting, indicating that solute-rich melt is difficult to flow through the solid network in mushy zone and the distance of solute-rich melt transport is short in the UDC casting process. As a result, the uniformity of the alloying elements’ distribution is improved in the UDC casting billet.

## 5. Conclusions

This paper represents the first time that UDC casting has been used to prepare a high alloying element Al–Zn–Mg–Cu ultra-high strength aluminum alloy billet. Compared with NDC casting, the quality of the billet prepared by UDC casting is dramatic improved. The effect of A-EMS and intercooling on microstructural refinement and composition homogenization have been investigated. Major conclusions are summarized as follows:

(1) With the help of A-EMS and intercooling, the grain size of the Al–Zn–Mg–Cu alloy billet was reduced from 520 ± 110 μm to 109 ± 10 μm. Macrosegregation of Al–Zn–Mg–Cu alloy billet was suppressed and the relative segregation of Zn, Mg and Cu is less than 3%.

(2) Explosive nucleation is responsible for grain refinement. Lots of nuclei originate from the graphite ring wall and in-mold cooler wall, and from survived fragments that were brought from the broken or fused dendrites. These nuclei are dispersed homogeneously throughout the sump and nuclei blocked the growth of each other in the limited space, resulting in a fine and uniform microstructure.

(3) The centrifugal force driven by A-EMS offset the temperature buoyancy force that is the driving force of the movement of melt from upper-periphery part towards the central part, and the permeability of the solute-rich melt flowing through the channels of the solid network was reduced by grain refinement. Therefore, melt flow in radial direction is impeded, resulting in less macrosegregation.

## Figures and Tables

**Table 1:** Casting process parameters of normal direct chill (NDC) and UDC.

Parameters	NDC	UDC
Pouring temperature (K)	1023	1023
Casting speed (mm min^−1^)	27	27
Cooling water (m^3^ h^−1^)	9	9
Electromagnetic current (A)	-	100
Electromagnetic frequency (Hz)	-	5
Cooler diameter (mm)	-	200
Cooling rate (W m^−2^ K^−1^)	-	300

## Data Availability

Data is contained within the article.
